# Protective Effect of Caffeic Acid Phenethyl Ester (CAPE) on Amiodarone-Induced Pulmonary Fibrosisin Rat

**Published:** 2011

**Authors:** Narjes Zaeemzadeh, Aliasghar Hemmati, Ardeshir Arzi, Mohammadtaha Jalali, Iran Rashidi

**Affiliations:** a *Department of Pharmacology and Toxicology, School of Pharmacy.*; b *Department of Laboratory Sciences, School of Paramedics, *; c *Department of Pathology, School of Medicine, Ahwaz Jundishapur University of Medical Sciences, Ahwaz, Iran.*

**Keywords:** Pulmonary fibrosis, Amiodarone, Caffeic acid phenethyl ester, Rat

## Abstract

Treatment with amiodarone, a commonly prescribed antidysrhythmic agent, is associated with pulmonary fibrosis (PF) which is a commonly progressive and untreatable disease. Caffeic acid phenethyl ester (CAPE) is a phenolic antioxidant and an active anti-inflammatory , anticancer, antimicrobial and antioxidant component of propolis (bee glue; a resinous hive product collected by honey bees). In the current study, the effects of CAPE on amiodarone-induced pulmonary fibrosis in rat were investigated.

Male rats were divided in to 4 groups. The first group only received amiodarone (6.25 mg/Kg) on first and third day. The second group received only vehicle (distilled water) with the same volume and in the same time as the first group. The third and fourth groups received amiodarone and were treated with CAPE , 5 and 10 µmol /day respectively, from 2 days before the first dose of amiodarone and until 21 days after the second dose of amiodarone. At the end of treatment course, lung tissue was removed for histopathology and biochemical evaluations. Malondialdehyde (MDA) concentration, myeloperoxidase MPO) and super oxide dismutase (SOD) activities were determined in lung tissue. Histopathological evaluation was performed using light microscopy. MDA level and the activity of myeloperoxidase and superoxide dismutase enzymes significantly decreased in the group which was treated with CAPE (5 µmol/Kg). However, 10 µmol/Kg CAPE had not such an effect. Both doses of CAPE could histopathologically reduce the fibrogenic effects of amiodarone . CAPE was shown to be effective in reducing amiodarone-induced pulmonary fibrosis with the dose of 5 µmol/Kg.

## Introduction

Pulmonary fibrosis (PF) is a progressive and essentially untreatable disease with a fatal outcome, which is characterized by an altered cellular composition of the alveolar region with excessive deposition of collagen. Typical features in this disease include dyspnea, diffuse interstitial infiltrates and poor prognosis. There are five million people worldwide that are affected by this disease. In the United States there are over 200,000 patients with pulmonary fibrosis. As a consequence of misdiagnosis, the actual number may be significantly higher. More than 40,000 of these patients expire annually. This is the same as the people who die from breast cancer. The pathogenesis of pulmonary fibrosis remains incompletely understood, but lung inflammation is a major underlying component of a wide variety of pulmonary fibroproliferative disorders (1-3).

It has been suggested in the last decades that the main responsible agent in PF is reactive oxygen species (ROS) such as superoxide, hydrogen peroxide, peroxynitrites and hydroxyl radicals, which are generated also in normal physiological conditions in the human body. These ROS are capable of initiating and promoting oxidative damage in the form of lipid per oxidation (LPO). The antioxidant serves as a defensive factor against free radicals in the body. Enzymes such as superoxide dismutase, catalase and glutathione peroxidase are the main system that opposes oxidation and act as a protective mechanism. If the free radicals production becomes more than the capacity of enzymatic system, the second line of defense (vitamins) may come to action. Antioxidant such as vitamins C and E quench free radicals and become oxidized and inactive (3-5).

Treatment with amiodarone (AM), commonly prescribed as an antiarhythmic agent, may decrease the incidence of mortality following myocardial infarction or due to out-of-hospital sudden cardiac arrest. However amiodarone administration is associated with few adverse effects including pulmonary injury resulting in fatal pulmonary fibrosis. The mechanisms for amiodarone pulmonary injury include direct toxicity to lung tissue, hypersensitivity reaction to amiodarone, and enhanced oxidative stress. Alteration of membrane properties and activation of alveolar macrophages and cytokine release are the other proposed mechanisms of amiodarone toxicity (6, 7).

Propolis or bee glue is a resinous hive product collected by honey bees from plant exudates (especially conifer buds) and contains more than 160 constituents. It is used to repair hive cracks by bees. Historically it has been used for various purposes, especially as a medicine and has been used empirically as a traditional remedy in folk medicine for centuries. Recently, propolis has been extensively marketed by the pharmaceutical industries as an alternative medicine and as the health-food in various parts of the world. Propolis has been claimed to improve the health and prevent diseases such as diabetes, heart diseases, and even cancer. Flavonoids are thought to be responsible for many of its biological and pharmacological activities including anticancer, anti-inflammatory, antimicrobial and antioxidant effects (7-9). Several investigations on propolis in Eastern Europe and South America have indicated that flavonoids concentrated in propolis are powerful antioxidants which are capable to scavenge free radicals (10).

Caffeic acid phenethyl ester (CAPE) is a phenolic antioxidant and is an active anti-inflammatory component of propolis. CAPE was shown to exert its antioxidant activity by suppressing lipid peroxidation, scavenging the reactive oxygen species, and inhibiting xanthine oxidase and nitric oxide synthase activities. It may reduce the activity of superoxide dismutase (11). It was also found to inhibit 5-lipoxygenase catalysed oxygenation of linoleic acid and arachidonic acid in micromolar (10 µM) concentrations and potently induces the inflammatory cell apoptosis through a glucocorticoid receptor independent mechanism (12) .

In the current study we investigated the anti-inflammatory and anti-oxidant effects of CAPE on amiodarone-induced pulmonary fibrosis in rat. The indices for evaluating these effects include: myeloperoxidase (MPO) activity, malondialdehide (MDA) concentration (indicators of oxidative stress), superoxide dismutase (SOD) activity (an antioxidant enzyme), and histopathological examination (12).

## Experimental

*Animals*

Male Wistar albino rats weighing 180-200 g were purchased from Animal house and research center, Jundishapur University of Medical Sciences, Ahwaz, Iran. The animals were kept on a standard food-pellet and tap-water ad libitum. The rats were housed in polycarbonate cages (5 animals per cage) and kept in an air-conditioned animals’ room at a temperature of 23 ± 3°C with a relative humidity of 50 ± 5%. The animals’ room was on a 12 h light-dark period cycle.

*Chemical agents*

Amiodarone: Donated by Amin Pharmaceutical Company, Isfahan, Iran Caffeic acid phenethyl ester (CAPE) (C8221): Purchased from Sigma-Aldrich, Germany

Malondialdehyde (MDA) assay kit (NWK-MDA01): Obtained from Northwest Life Science Specialties, LLC (NWLSS™), Vancouver, Canada

Myeloperoxidase (MPO) assay kit (ASA-OO1): Obtained from Cytostore Targeted Research Tools, Canada 

Superoxide dismutase (SOD) assay kit (FR10): Obtained from Oxford Biomedical Research, USA.

*Induction of pulmonary fibrosis by amiodarone*

Amiodarone was dissolved in distilled water at 60°C and allowed to be cooled in room temperature before instillation(13). The rats were anesthetized with sodium methohexital (40 mg/Kg). Two doses of amiodarone (6.25 mg/Kg) were givenas intra-tracheal instillations, first on what is designated as day 1 and the second on day 3 (14).

*Drugs treatment groups*

The animals divided into the following groups (10 rats per group (n = 10)): Group 1: This group received only intra-tracheal amiodarone as positive control. Group 2: The rats in this group received only intra-tracheal vehicle (distilled water) as negative control. Group 3: This group received CAPE 5 μmol/Kg/day, IP, from 2 days before the first dose of amiodarone instillation until 3 weeks after the second dose of amiodarone instillation. Group 4: Received CAPE 10 μmol/Kg/day, IP, 2 days before the first dose of amiodarone instillation until 3 weeks after the second dose of amiodarone (15). The vehicle in all solutions was distilled water.

*Collecting tissue samples*

After the last injection of CAPE, rats were killed by sternotomy, following deep ether-induced anesthesia. After mid-line sternotomy, both lungs were removed. Right lungs were preserved in formaldehyde solution (10%) for histopathological evaluation and left lungs were divided into 3 parts, washed with heparinized saline and stored in -70°C for biochemical examinations. 

*Biochemical assays*

a) *Malondialdehyde *(MDA) assay: This assay is based on the reaction of MDA with thiobarbituric acid (TBA); forming a MDA-TBA2 adduct that absorbs at 532 nm (16, 17). The assay was performed through the above mentioned kit and via the manufacturer instructions.

b) *Myeloperoxidase* (MPO) assay: In this assay, H_2_O_2_ is broken down by MPO released from samples by homogenization in detergent. The produced radical oxygen (O) combines with a hydrogen-donating chromogen which is thereby converted to a colored compound. The appearance of this colored compound is measured, over time, with a spectrophotometer (at 450 nm) to determine MPO activity in a sample (18). The assay was performed through the above mentioned kit and via the manufacturer protocol.

c) *Superoxide dismutase* (SOD) assay: The assay’s method is based on the SOD-mediated increase in the rate of autoxidation of 5, 6, 6a, 11btetrahydro-3, 9, 10-trihydroxybenzo[c] fluorene in aqueous alkaline solution to yield a chromophore with maximum absorbance at 525 nm. The chromophore has not been isolated or characterized (19). The assay is also performed through the above mentioned kit and via the manufacturer protocol.

*Histopathological examination* *and scoring*

Lung tissues were fixed in 10% formaldehyde solution for paraffin slides and sectioned at approximately 5 μm thickness. Tissue affixed to a glass slide deparaffinized, rehydrated and counterstained with hematoxylin and eosin (H&E) (20). The slides were examined by light microscopy and photographed (20).According to the literature, the following scoring codes were used: 

0 = Normal lung tissue; 1 = Minimal fibrous with thickening of alveolar or bronchial wall; 2 = Moderate thickening of bronchial wall with mild damage to lung architecture; 3 = Severe distortion of lung structure and presence of large fibrous areas (21).

*Statistical analysis*

Statistical comparison was made by one-way ANOVA. Significant f- values were tested with Tukey’s test. Data are presented as mean ± SEM.

## Results

*MDA concentrations in lung tissue*

**Figure 1:**
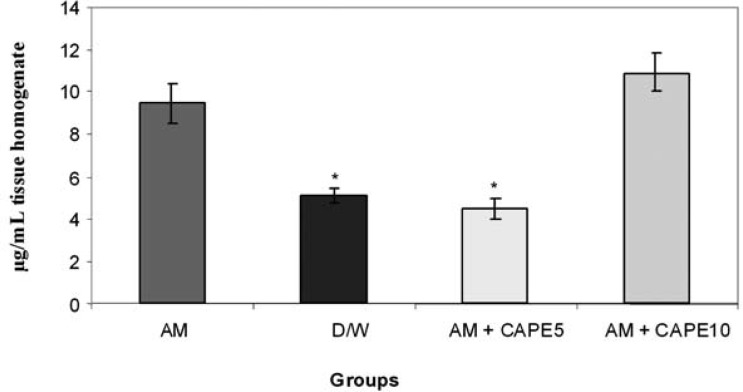
lung tissue Malondial dehyde concentrations (µg/ml tissue homogenate) significant difference between amiodar one- treated group and negative control or 5 µM CAPE- treated groups are indicated by *(p<0.001).

CAPE (5 µM) could significantly (p < 0.001) reduce the MDA concentration in amiodarone-treated group. However, higher concentration of CAPE (10 µM) increased the MDA in amiodarone-treated lung tissue (Figure 1).

*MPO activity in lung tissue*

**Figure 2:**
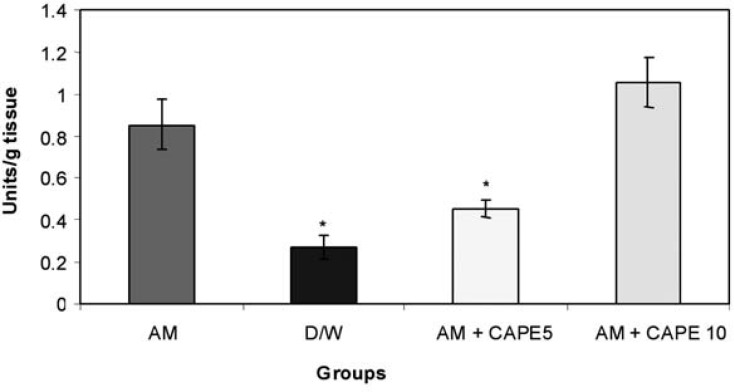
lung tissue Myeloperoxidase (units/g tissue) Significant differences between amiodarone-treated group negative control of 5 µM CAPE-treated groups are indicated by *(p<0.001).

CAPE (5 µM) could significantly (p<0.001) reduce the MPO activity in amiodarone -treated group . However, higher concentration of CAPE (10 µM) increased the MPO activity in amiodarone -treated lung tissue (Figure 2).

*SOD activity in lung tissue*

**Figure 3:**
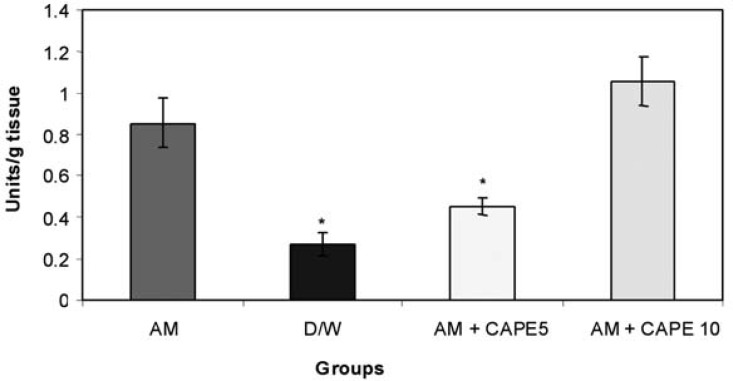
lung tissue superoxide dismutase (SOD) activity (units/ml tissue homogenate) Significant differences between amiodarone-treated group and negative control or 5 µM CAPE-treated groups are indicated by *(p<0.001).

 CAPE (5 µM) could significantly (p < 0.001) reduce the SOD activity in amiodarone – treated group. However, higher concentration of CAPE (10 µM) could not reduce the SOD activity in amiodarone – treated lung tissue (Figure 3).

*Light microscopic results*

**Figure 4:**
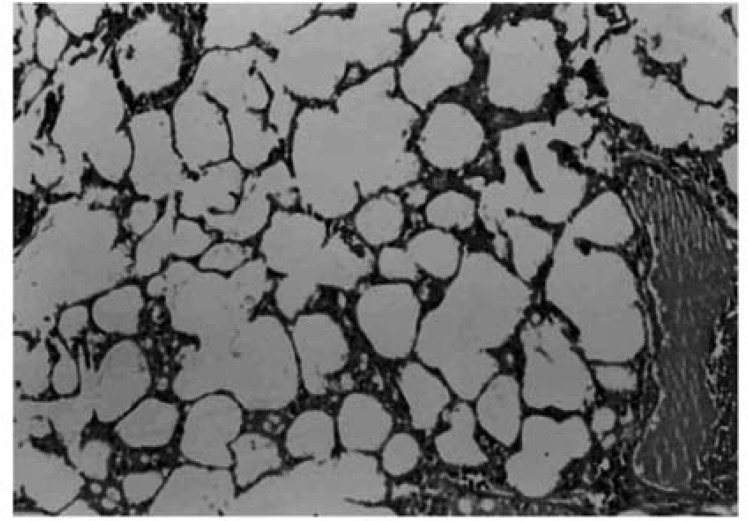
Photomicrograph of negative control lung tissue which received only IT distilled water, showing normal lung stracture. No inflammatory cells are evident. Donated score = 0 (H&E, ×100).

 Histopathological evaluation of the pulmonary tissue was performed with light microscopy for the 4 different experimental groups after the end of study course. Lungs of rats in negative control group, which received distilled water, showed normal lung structure and no lesion was obvious (Figure 4). 

**Figure 5:**
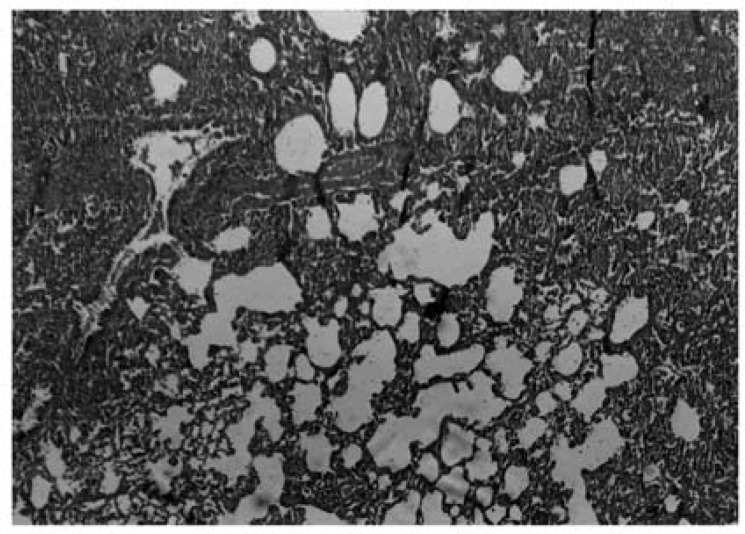
Photomicrograph of rat lung tissue 3 weeks after the second amiodarone administration. There is an increase in cellurality of alveolar septum and intra – alveolar fibrosis with collagenous bands accompanying great septal thickness and diffuse damage to lung architecture is observed. Donated score is 3 (H&E, ×100).

Three weeks after these condamiodaronein stillation, there were in filtration of in flammatory cells and fibro blast and myofibroblast proliferation associated with perivascular and peribronchial fibrosis (Figure5).

**Figure 6:**
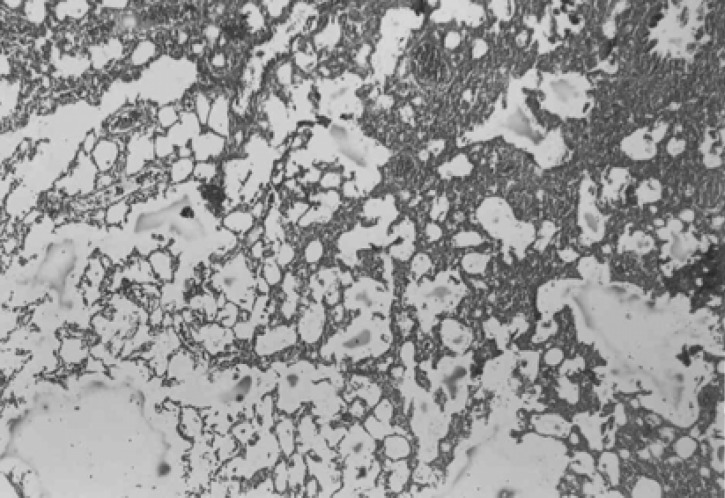
Photomicrograph of rat lung tissue which received amiodaron e+ CAPE 5µmol/kg . less interstitial fibrosis with scattered septal thichness and slight infiltration of lymphocytes in comparison to positive control group is seen. Donated score = 2 (H&E, ×100).

**Figure 7:**
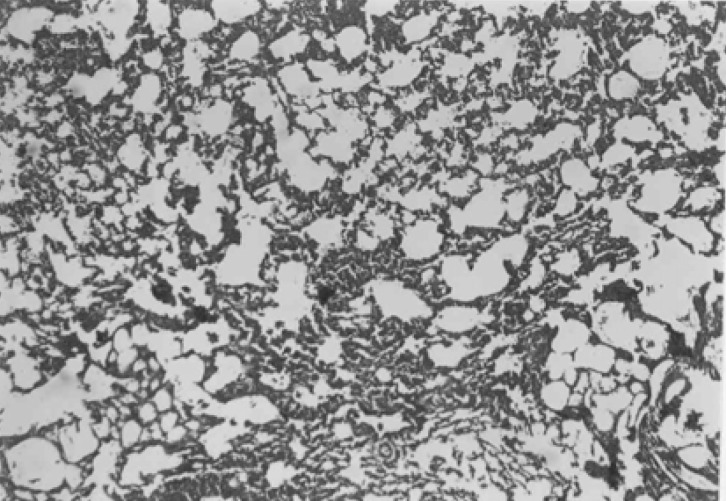
Photomicrograph of rat lung tissue which received amiodaron e+ CAPE 10µmol/kg . Decreased fibrosis and septal thickness is evident. Donated score = 1 (H&E, ×100).

 In both CAPE-treated groups, considerable improvement in tissue structure was observed. In these groups, less inflammatory cells and decreased alveolar thickening was evident (Figures 6 and 7).

## Discussion

Pulmonary fibrosis (PF) isachronic, often fatal inflammatory interstitial lung disorder characterized by an accumulation of alveolar macrophages and neutrophilsin the lower respiratory tract , parenchymal cell injury, and fibrosis of the alveolar walls (22). 

The evidence of a redox imbalance in lung fibrosisis substantial, and the rationale for testing antioxidants as potential new therapeutics for lung fibrosis is appealing. Current animal models of lung fibrosis showed a clear involvement of ROS in their pathogenesis (23).

The mechanisms proposed for amiodarone –induced PF are: (i) altered inflammatory mediator release; (ii) cell membrane perturbation; (iii) phospholipidosis promotion; (iv) altered Ca^2+^cellular homeostasis; (v) mitochondrial dysfunction; and (vi) free radical production. Although the exact mechanism is not fully understand (24).

In this model of amiodarone-induced lung injury we observed a decrease in malondialdehyde concentrations, and myeloperoxidase and superoxide dismutase activities in lung tissue with CAPE (5 μmol/kg). In addition, in histopathological examination there were structural changes in amiodarone- treated group in comparison to negative control group (D/W treated animals). A pronounced decreased in lung fibrosis was occurred in CAPE treated groups. Previous researches showed free radical scavenging, anti-inflammatory, anti-fibrotic and anti-endotoxine activities for CAPE (12,21,25).

In this study MDA increased significantly in the amiodarone treated group in comparison with the vehicle treated rats, and CAPE with the dose of 5 μmol/kg could decrease it significantly even less than the negative control group . As MDA is a highly reactive agent produced as a by product of poly unsaturated fatty acid peroxidation and arachidonic acid metabolism, (26) we can say that low- dose CAPE is a very potentanti –lipid peroxidation and antioxidant agent. In contrast to previous studies (12,21), there was not any improvement with 10 μmol/kg CAPE and event here was significant increase in MDA concentration in comparison with negative control and low dose CAPE groups. 

In current study we also found a significant decrease in MPO activity in the low-dose CAPE treated animals in comparison to positive control. When accumulated and activated, neutrophils can release myeloperoxidase , an 115,000-dalton protein which can interact with H_2_0_2_ , a product of both mono nuclear phagocytes and neutrophils, to form a highly toxic anion, which can initiate oxidative stress (12,22). Our results suggest that low dose CAPE could successfully decrease inflammation and oxidant- mediated lung toxicity . However; high dose of CAPE had opposite effect compared to the previous studies; In fact there was significant increase in MPO activity in comparison with negative control and low dose CAPE groups (12,21).

According to these findings it may exists a toxic effect for CAPE of 10μmol/kg via oxidant system. One possible explanation for this finding is that administration of high dose of CAPE (10 μmol/kg) might have caused tissue injury related to another species of active oxygen. It should evaluate more, as the other studies do not show such an effect. Also it may be an observer error or a technical defect in some studies.

We also found significant increase in SOD activity in positive control group in comparison with negative control. It is reasonable, as in the normal airway mucosa, anti oxidant defense systems, such as superoxide dismutase (SOD), glutathione peroxidase, and catalase exist to protect the mucosa from various oxidant stimuli and in fact this enzyme has applied for the treatment of PF in some studies (27,28) and they showed inhibitory effects of SOD on bleomycine –induced pulmonary fibrosis. As it was expected there was significant decrease in SOD activityin 5 μmol/kg CAPE –treated group, close to negative control, which shows 5 μmol/kg CAPE decreased the need for compensatory increase in SOD activity against amiodarone –induced oxidative injury. 

In this study, we concluded that systemic CAPE with the dose of 5μmol/kg can diminish amiodarone-induced lung injury successfully. Therefore; CAPE may be a potential treatment for pulmonary fibrosis. However more investigations are required to elucidate the exact mechanism of this novel agent in pulmonary fibrosis. 
